# Aberrant expression of HDL-bound microRNA induced by a high-fat diet in a pig model: implications in the pathogenesis of dyslipidaemia

**DOI:** 10.1186/s12872-021-02084-5

**Published:** 2021-06-06

**Authors:** Guoyuan Sui, Lianqun Jia, Nan Song, Dongyu Min, Si Chen, Yao Wu, Guanlin Yang

**Affiliations:** 1 Key Laboratory of Ministry of Education for Traditional Chinese Medicine Viscera-State Theory and Applications, Liaoning University of Traditional Chinese Medicine, Shenyang, Liaoning People’s Republic of China; 2 The Affiliated Hospital of Liaoning University of Traditional Chinese Medicine, Shenyang, Liaoning People’s Republic of China

**Keywords:** HDL, miRNA, Dyslipidaemia, High-fat diet

## Abstract

**Background:**

A high-fat diet can affect lipid metabolism and trigger cardiovascular diseases. A growing body of studies has revealed the HDL-bound miRNA profiles in familial hypercholesterolaemia; in sharp contrast, relevant studies on high-fat diet-induced dyslipidaemia are lacking. In the current study, HDL-bound miRNAs altered by a high-fat diet were explored to offer some clues for elucidating their effects on the pathogenesis of dyslipidaemia.

**Methods:**

Six pigs were randomly divided into two groups of three pigs each, namely, the high-fat diet and the balanced diet groups, which were fed a high-fat diet and balanced diet separately for six months. HDL was separated from plasma, which was followed by dissociation of the miRNA bound to HDL. miRNA sequencing of the isolated miRNA was performed to identify the differential expression profiles between the two groups, which was validated by real-time PCR. TargetScan, miRDB, and miRWalk were used for the prediction of genes targeted by the differential miRNAs.

**Results:**

Compared with the balanced diet group, the high-fat diet group had significantly higher levels of TG, TC, LDL-C and HDL-C at six months. miRNA sequencing revealed 6 upregulated and 14 downregulated HDL-bound miRNAs in the high-fat diet group compared to the balanced diet group, which was validated by real-time PCR. GO enrichment analysis showed that dysregulated miRNAs in the high-fat diet group were associated with the positive regulation of lipid metabolic processes, positive regulation of lipid biosynthetic processes, and positive regulation of Ras protein signal transduction. Insulin resistance and the Ras signalling pathway were enriched in the KEGG pathway enrichment analysis.

**Conclusions:**

Twenty HDL-bound miRNAs are significantly dysregulated in high-fat diet-induced dyslipidaemia. This study presents an analysis of a new set of HDL-bound miRNAs that are altered by a high-fat diet and offers some valuable clues for novel mechanistic insights into high-fat diet-induced dyslipidaemia. Further functional verification study using a larger sample size will be required.

**Supplementary Information:**

The online version contains supplementary material available at 10.1186/s12872-021-02084-5.

## Background

Dyslipidaemia is a disease characterized by elevated levels of total cholesterol (TC), low-density lipoprotein cholesterol (LDL-C), and triglycerides (TG) and lower levels of high-density lipoprotein cholesterol (HDL-C) compared to normal conditions [1]. The morbidity of dyslipidaemia is 34%-60% worldwide [2–4]. Dyslipidaemia is a risk factor for cardiovascular disease (CVD), accounting for 31% of global disease deaths, and is the leading cause of disease death worldwide [5–7]. For the above mentioned reason, it is critical to explore the pathogenesis of dyslipidaemia for the prevention of CVD. Although many studies have reported that a high-fat diet promotes the development of dyslipidaemia [8], the underlying molecular processes behind its effects are still poorly understood.

MicroRNAs (miRNAs) are a class of endogenous non-coding RNAs consisting of 19–24 nucleotides. miRNAs regulate gene expression post-transcriptionally and participate in many physiological and pathological processes, including inflammation, lipid metabolism, apoptosis, and angiogenesis [9–15]. It is recognized that miRNAs are unstable in the extracellular environment; however, circulating in the blood with the aid of exosomes, microparticles, lipoproteins or protein complexes makes them highly stable [16, 17]. High-density lipoprotein (HDL)-bound miRNAs may act as markers for the occurrence and development of various diseases [18, 19]. It was reported that dramatic alterations in HDL-bound miRNAs were observed in patients with familial hypercholesterolaemia, atherosclerosis, angina, myocardial infarction, unstable coronary artery disease and acute coronary syndrome [18–20]; for example, HDL-bound miR-223, HDL-bound miR-24, and HDL-bound miR-486 are increased and HDL-bound miR-92a is decreased in patients with familial hypercholesterolaemia [18]. Abnormalities in HDL-bound miR-486 and HDL-bound miR-92a may increase the risk of coronary artery disease [19]. Transportation of miRNAs carried by HDL to recipient cells regulates gene expression, which is involved in physiological processes such as lipid metabolism and inflammation [21, 22]. Thus, characterizing the high-fat diet-induced changes in the expression of HDL-bound miRNA is key to understanding the molecular mechanisms underlying dyslipidaemia. However, the impact of a high-fat diet on HDL-bound miRNA expression profiles and the putative role of these HDL-bound miRNAs in high-fat diet-induced dyslipidaemia are not fully understood. Pigs are an ideal disease model in medical research because of their evolutionary conservation and genetic similarity compared to humans [23]. For this reason, the study of HDL-bound miRNAs in pigs fed a high-fat diet will be of significance for understanding the pathogenesis of high-fat diet-induced dyslipidaemia.

In this study, we identified a new set of HDL-bound miRNAs whose expression profiles were significantly altered by a high-fat diet, followed by in-depth bioinformatics analysis. Our study may offer some valuable clues for novel mechanistic insights into high-fat diet-induced dyslipidaemia.

## Methods

### Samples

The research protocol was approved by the Ethics Committee of Beijing Rixin Technology Co., Ltd. (RXKJ-IACUC-2018006). The sample size was estimated according to previous studies [24, 25]. Six male Bama minipigs (24 weeks old, 20–25 kg) were purchased from Tong He Sheng Tai Company (Beijing, China) and raised in controlled conditions (temperature 22 ± 1 °C, humidity 50 ± 5%). Using the random number table method, six pigs were randomly divided into two groups of three pigs each: the balanced diet and high-fat diet groups. The balanced diet group was fed a balanced diet (48% corn, 20% wheat flour, 15% soybean cake, 12% rice bran, 5% fish meal), and the high-fat diet group was fed a high-fat diet (6% peanut oil, 6% lard, 3% cholesterol, 1% cholate and 84% regular balanced diet). Peripheral blood was sampled after zero months and six months of feeding, and serum and plasma were separated for further study [25, 26]. The reason for selecting 0 months is that we wanted to show the equilibrium of the balanced diet group and high-fat diet group at the beginning of the experiment. Pigs were anaesthetized using intramuscular injection of Zoletil 50 (20 mg/kg). The pigs were sacrificed when unconscious using the blood-letting method [27]. The person responsible for data collection and analysis was unaware of the group assignments.

### Lipid measurement

The lipid profile was analysed with an automatic biochemical analyser (SIEMENS, Germany). TG, TC, LDL-C, and HDL-C in the serum were analysed using corresponding kits (all from Siemens Healthcare Diagnostics Products GmbH, Marburg, Germany).

### HDL isolation

HDL isolation was based on a previous study [28]. Briefly, sequentially centrifuge was performed for blood samples to obtain plasma free of red/white blood cells and platelets. The exosomes with a density similar to HDL were removed from plasma by using ExoQuick solution (Invitrogen, America) before HDL isolation. HDL was isolated by a 3-step density gradient ultracentrifugation process employing an ultracentrifugee. To avoid interference with subsequent experiments, excessive salt added during density gradient ultracentrifugation was removed using centrifugal filter devices.

### RNA extraction

miRNAs were separated from purified HDL with a plasma miRNA isolation and purification kit following the manufacturer’s instructions (QIAGEN, Germany) and a previous study [28]. The RNA concentration and purity were quantified by a NanoDrop ND-1000, and samples with A260/A280 values within 1.8–2.1 were used for the following study.

### Sequencing process and analysis

The construction of the miRNA library and sequencing were performed by Cloudseq Science and Technology Corp., Ltd. (Shanghai, China). The whole miRNA library was constructed with Ribo-Zero rRNA removal kits (Illumina, San Diego, CA, USA) and the NEB small RNA library kit for Illumina (New England Biolabs, Ipswich, MA, USA) following the manufacturer’s instructions. Reads sequenced were aligned with the pre-miRNA database after quality control with base calling confidence no less than Q30, and MiRDeep2 was used to predict novel miRNAs. Differentiated miRNAs with a log2 fold change larger than 1 and *p*-value less than 0.05 between two groups were considered significant.

### Target mRNA of selected miRNAs

TargetScan (http://www.targetscan.org), miRDB (https://mirdb.org) and miRWalk (http://mirwalk.umm.uni-heidelberg.de/) were used to predict potential targets of differentially expressed miRNAs, and targets of miRNAs predicted by at least two databases were used for further analysis.

### GO and KEGG pathway analyses

GO and KEGG enrichment analyses were performed to reveal the function of target genes regulated by differentiated miRNAs, with *p*-values and q-values less than 0.05.

### Validation of the miRNA sequencing results by real-time PCR

cDNA was synthesized with M-MLV reverse transcriptase (Invitrogen, Carlsbad, CA, USA) after extraction of RNA, followed by real-time PCR using qPCR SYBR Green master mix (CloudSeq, Shanghai, China) according to the manufacturer’s instructions. U6 was set as the reference. The 2^−ΔΔCt^ method was used to calculate the relative expression levels of selected miRNAs. The sequences of the primers are shown in Additional file 1: Table S1.

### Statistical analysis

All continuous variables are presented as the mean ± standard deviation of the mean. If data were normally distributed, *t*-tests were used to analyse the difference between the high-fat diet group and balanced diet group; if not, the Mann–Whitney *U* test was used. A *p* value of less than 0.05 was considered statistically significant. Analyses were performed using SPSS version 13.0.

## Results

### Lipid profile results

**Fig. 1:**
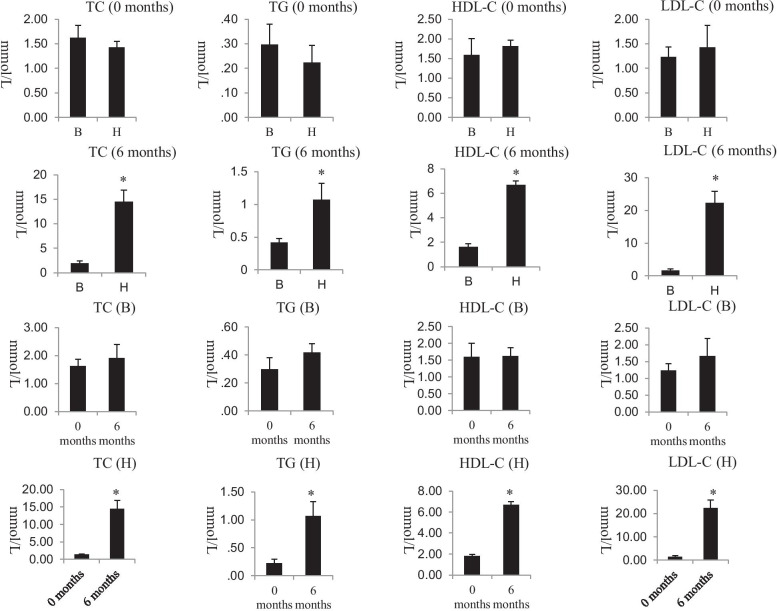
Characteristics of the lipid profile are shown in detail (H: high-fat diet group, B: balanced diet group; *: *p* < 0.05)

There was no significant difference in the lipid profile between the balanced diet group and the high-fat diet group at 0 months; compared with the balanced diet group, the high-fat diet group had significantly higher levels of TG, TC, LDL-C, and HDL-C at six months (*p* < 0.05). In the balanced diet group, there was no significant difference in the lipid profile between 0 and 6 months. In the high-fat diet group, compared to 0 months, TG, TC, LDL-C, and HDL-C significantly increased at six months (*p* < 0.05) (Fig. 1).

### Sequencing results and characteristics of miRNAs

**Fig. 2:**
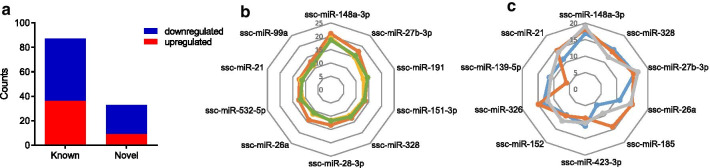
Characteristics of miRNAs shown in detail. **a** Bar chart showing the number of whole miRNAs. **b** Radar plot presenting the most significant miRNAs in the high-fat diet group, which are shown in the form of normalized counts. H, high-fat diet group. **c** Radar chart exhibiting the most significant known miRNAs in the balanced diet group, B, the balanced diet group

A total of 341 miRNAs were generated after raw data analysis, among which a cohort of 121 miRNAs were identified consisting of 87 known and 34 novel miRNAs (Fig. 2a). Forty-seven miRNAs were upregulated and 74 downregulated. We found that ssc-miR-148a-3p, ssc-miR-27b-3p and ssc-miR-191 were the most significantly detected miRNAs in the high-fat diet group (Fig. 2b). In the balanced diet group, ssc-miR-148a-3p, ssc-miR-328 and ssc-miR-27b-3p were the most significantly detected miRNAs (Fig. 2c).

### Differentially expressed miRNAs

**Fig. 3:**
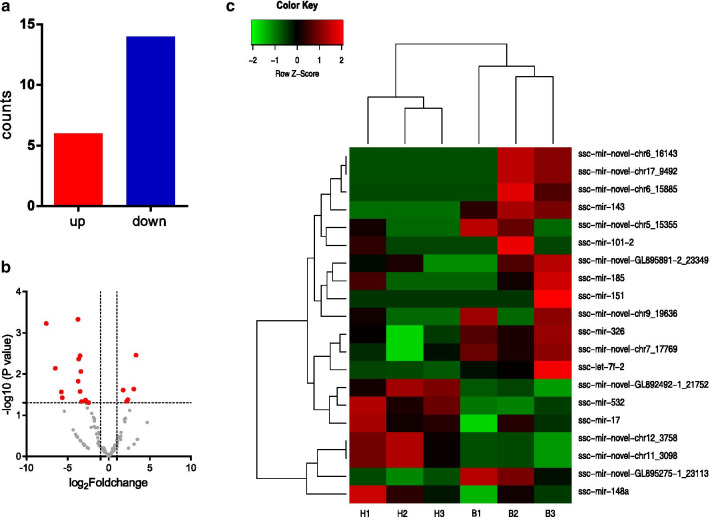
Differentially expressed miRNAs. **a** Counts of differentially expressed miRNAs. Red bars, upregulated miRNAs; blue bars, downregulated miRNAs. **b** Significantly differentially expressed miRNAs. Vertical lines denote the twofold change, and horizontal lines indicate a *p*-value of 0.05. Red points show the differentially expressed miRNAs with statistical significance. **c** Clustering analysis of all differentially expressed miRNAs. Heatmap drawn showing miRNAs with altered expression levels greater than twofold change and *p*-values less than 0.05. H: high-fat diet group; B: balanced diet group

In total, 20 differentially expressed miRNAs with fold changes larger than 2 and *p*-values less than 0.05 were identified, among which 6 were upregulated and 14 were downregulated (Fig. 3a). Differentiated miRNAs with statistical significance are shown in a volcano plot (Fig. 3b) and by clustering analysis (Fig. 3c).

### GO analysis

**Fig. 4:**
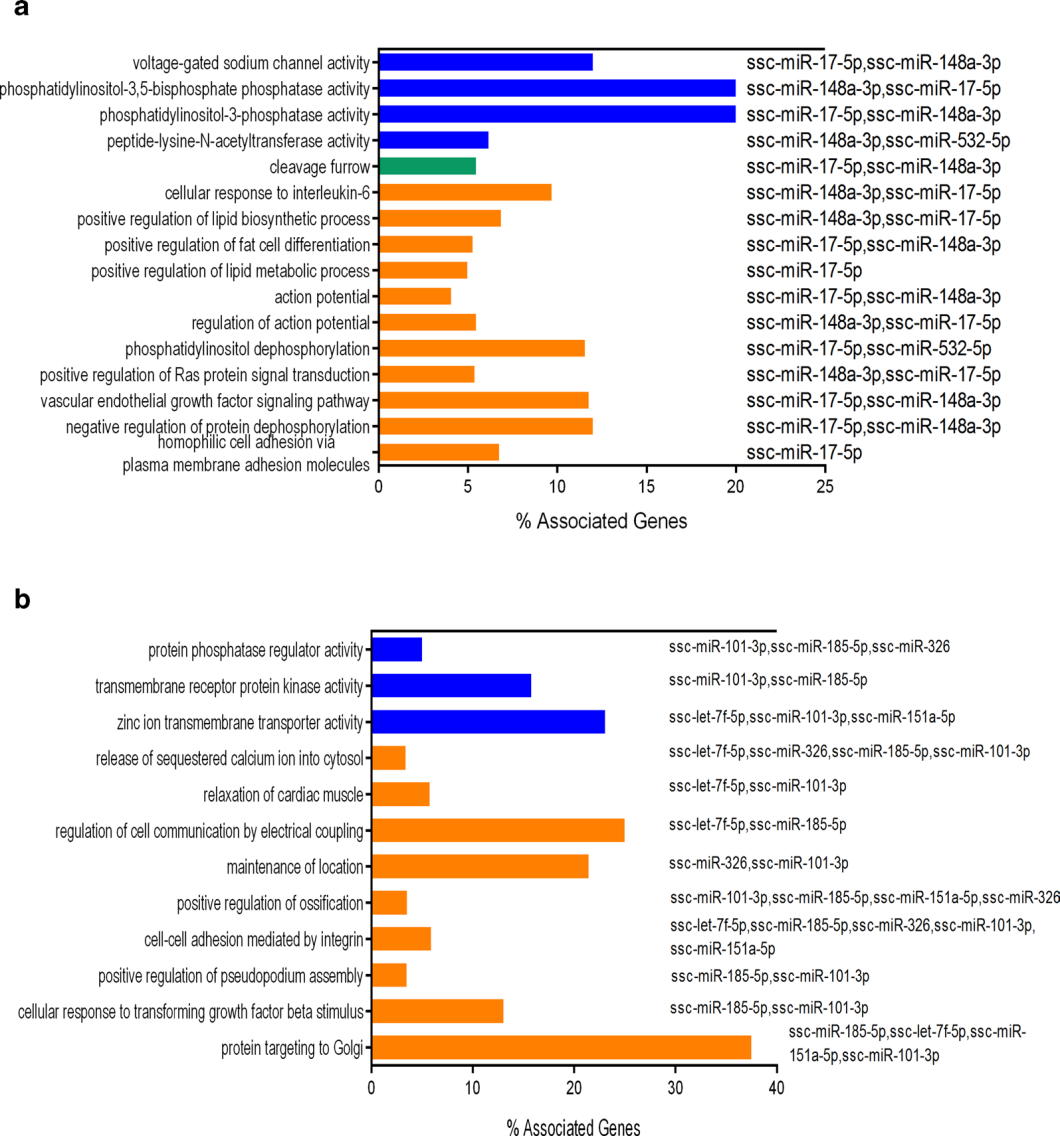
GO analysis of differentially expressed miRNAs. **a** GO annotation of upregulated genes in biological processes, cellular components and molecular functions. **b** GO annotation of downregulated genes in biological processes and molecular functions. Blue bars, molecular functions; green bars, cellular components; orange bars, biological processes. All enriched terms with *p*-values less than 0.05. The left Y-axis represents the enriched terms; the X-axis indicates associated genes

To study the underlying mechanism by which differentiated miRNAs play a role in the regulation of gene expression, GO enrichment analysis was performed. The results are shown in Fig. 4. The results revealed that 173 genes may be potential targets of the upregulated miRNAs ssc-miR-148a-3p, ssc-miR-17-5p, and ssc-miR-532-5p. In terms of biological processes, several processes, including negative regulation of protein dephosphorylation, positive regulation of Ras protein signal transduction, positive regulation of lipid metabolic processes, and positive regulation of lipid biosynthetic processes, were mainly enriched. Peptide-lysine-*N*-acetyltransferase activity, phosphatidylinositol-3-phosphatase activity, and phosphatidylinositol-3,5-bisphosphate phosphatase activity were enriched in the molecular function category. A total of 212 genes may be potential targets of the downregulated miRNAs, including ssc-let-7f-5p, ssc-miR-101-3p, ssc-miR-143-3p, ssc-miR-151-5p, ssc-miR-185-5p, and ssc-miR-326. Proteins targeting the Golgi, cellular response to transforming growth factor beta stimulus and positive regulation of pseudopodium assembly were the main enriched biological processes. Regarding molecular function, peptide-lysine-*N*-acetyltransferase activity, phosphatidylinositol-3-phosphatase activity and phosphatidylinositol-3,5-bisphosphate phosphatase activity were enriched.

### KEGG analysis

**Fig. 5:**
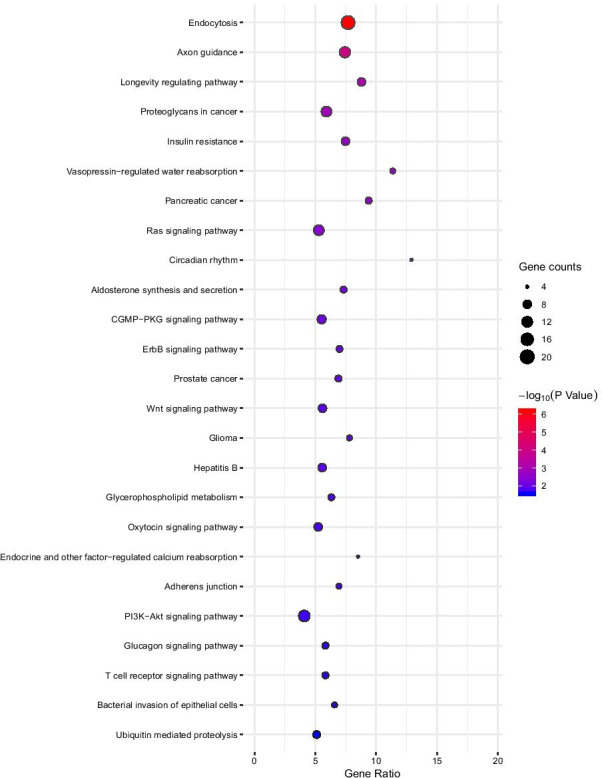
KEGG analysis of differentially expressed miRNAs. The enrichment factor represents the ratio between the differentially expressed genes and all annotated genes enriched in the pathway. The size of the bubble denotes the number of different genes; colour depth indicates the *p*-value

Analysis revealed that all targeted genes were enriched in 38 pathways, including endocytosis, insulin resistance, the Ras signalling pathway, the ErbB signalling pathway, the Wnt signalling pathway, glycerophospholipid metabolism, and the phosphatidylinositol 3-kinase (PI3K)-Akt signalling pathway. The top 25 enriched KEGG pathways are shown in Fig. 5.

### Validation of the miRNAs

**Fig. 6:**
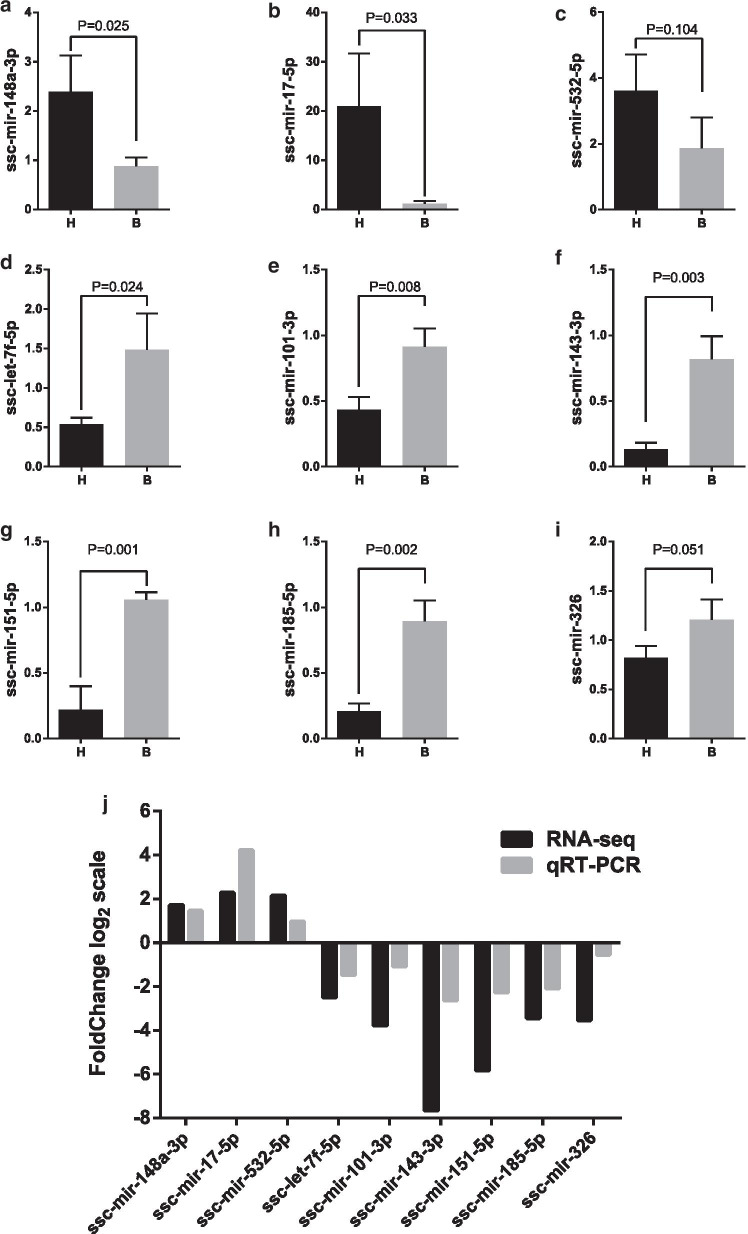
The quantitative real-time PCR validation of the relative expression levels of selected miRNAs. **a**–**i** The expression of selected RNAs was validated by qRT-PCR in tissues of the high-fat diet group and balanced diet group. Data are shown as the mean ± SD. **j** Comparison of qRTPCR and RNA sequencing results. The vertical axis shows the fold change (log2 scale) of each miRNA. H: high-fat diet group; B: balanced diet group

Real-time PCR was performed to verify the sequencing results, and it validated the expression trend of 9 miRNAs among the top dysregulated miRNAs, which was in line with the sequencing results (Fig. 6).

## Discussion

HDL may transport lipids, proteins, and miRNAs. HDL-bound molecules may play roles in cardiovascular protection and serve as targets of novel therapeutic agents [18, 19]. Transportation of miRNAs carried by HDL to recipient cells regulates gene expression, which is involved in physiological processes such as lipid metabolism and inflammation [21, 22]. Our study was the first to reveal aberrant expression of HDL-bound miRNA induced by a high-fat diet, and we identified 20 dysregulated HDL-bound miRNAs, including 6 upregulated and 14 downregulated miRNAs. The result was validated by real-time PCR. A high-fat diet altered the HDL-transported miRNA signature. These differentially expressed HDL-bound miRNAs may be involved in high-fat diet-induced dyslipidaemia. A previous study reported that 22 HDL-bound miRNAs were significantly dysregulated in familial hypercholesterolaemia [19]. The dysregulated expression profiles of HDL-bound miRNA in familial hypercholesterolaemia in this study differed from those of high-fat diet-induced dyslipidaemia in our study, which implied that there are different metabolic mechanisms in familial hypercholesterolaemia and high-fat diet-induced dyslipidaemia.

In our study, HDL-bound miR-17-5p and HDL-bound miR-148a-3p were upregulated in the high-fat diet group. The most dramatic alteration in expression levels was HDL-bound miR-17-5p. It was reported that miR-17-5p bound to HDL particles was decreased in overweight and obese subjects, which coincided with our study [29]. miR-148a was positively associated with LDL-C and TG levels in humans [30–32]. miR-17-5p and miR-148a-3p were reported to regulate lipid metabolism in the liver [33, 34]. In addition, we found that HDL-bound miR-143-3p and HDL-bound miR-185-5p were downregulated in the high-fat diet group. miR-143-3p was reported to exhibit negative correlations with TC and LDL-C [35]. miR-185-5p expression was decreased in C57BL/6J mice fed a high-fat diet [36]. It affects cholesterol and fatty acid metabolism in hepatic cells [31]. Scavenger receptor type B1 (SR-B1) is involved in the selective uptake of cholesteryl ester from HDL. SR-B1 is highly expressed in the liver and some cells, such as endothelial cells, monocytes/macrophages, smooth muscle cells, adipocytes, and steroidogenic cells [21, 22, 37, 38]. The capability of HDL to deliver miRNAs to recipient cells via SR-B1 has been found in macrophages, endothelial cells, and hepatocytes [21, 22, 37, 38]. These reports collectively showed that HDL-bound miR-148a-3p, HDL-bound miR-17-5p, HDL-bound miR-143 and HDL-bound miR-185 may participate in the pathogenesis of high-fat diet-induced dyslipidaemia.

The lack of studies on the function of HDL-bound miRNAs was the main impediment for further research on the regulatory role of HDL-bound miRNAs; therefore, we predicted the function of HDL-miRNAs by performing bioinformatic analysis on these potential gene targets. We filtered the targets of dysregulated miRNAs by combined prediction with three databases: TargetScan, miRDB, and miRWalk. GO analyses of these screened mRNAs revealed that positive regulation of lipid metabolic processes, positive regulation of lipid biosynthetic processes, and positive regulation of Ras protein signal transduction were the main enriched terms. KEGG pathway analyses showed that these mRNAs were mainly associated with insulin resistance and the Ras signalling pathway. In the present study, dysregulated miR-17-5p and miR-148-3p were found to target potential genes, such as cAMP-response element binding protein (CREB1), lysophosphatidylglycerol acyltransferase 1 (LPGAT1) and peroxisome proliferator activated receptor gamma coactivator 1 alpha (PPARGC1A), which regulate lipid biosynthetic and metabolic processes. CREB regulates transcription coactivator 2 (CRTC2) and regulates mTOR-mediated lipid homeostasis in the liver [39]. LPGAT1, which is most highly expressed in the liver, plays a significant role in hepatic triacylglycerol synthesis and secretion [40]. PGC-1α (encoded by PPARGC1A) was reported to regulate adaptive metabolism [41]. HDL-bound miR-17-5p, HDL-bound miR-148-5p, HDL-bound miR-532-5p, HDL-bound miR-143-3p, HDL-bound miR-185-5p, and HDL-bound miR-326 have been found to affect insulin resistance, and insulin resistance may lead to glucose and lipid metabolism disorders [42]. HDL-bound miR-17-5p, HDL-bound miR-532-5p, HDL-bound miR-143-3p and HDL-bound miR-185-5p were involved in the Ras signalling pathway, and KRAS could alleviate high-fat diet-induced non-alcoholic fatty liver disease by inhibiting the synthesis and promoting beta-oxidation of fatty acids [43]. These results supported the hypothesis that positive regulation of lipid metabolic processes, positive regulation of lipid biosynthetic processes, positive regulation of Ras protein signal transduction, insulin resistance and the Ras signalling pathway are involved in the pathological process of dyslipidaemia. Knowledge of HDL-bound miRNA alterations induced by a high-fat diet will deepen our understanding of the pathogenesis of dyslipidaemia.

### Limitations

Our study had some limitations. First, the sample size was small. In future studies, we will further increase the sample size to verify our results. Second, functional analyses are needed to validate the results obtained here and to better elucidate the role of these molecules in the pathogenesis of high-fat diet-induced dyslipidaemia.

## Conclusions

Basically, our study showed that 20 HDL-bound miRNAs are significantly dysregulated in high-fat diet-induced dyslipidaemia. HDL-bound miR-148a-3p and HDL-bound miR-17-5p were upregulated, and HDL-bound miR-143 and HDL-bound miR-185 were downregulated in high-fat diet-induced dyslipidaemia. GO and KEGG analyses contributed to the analysis of the biological function of the candidate differentially expressed HDL-bound miRNAs. The results presented an analysis of a new set of HDL-bound miRNAs that are altered by a high-fat diet and offer some valuable clues for novel mechanistic insights into high-fat diet-induced dyslipidaemia. Further functional verification study using a larger sample size will be required.

## Supplementary Information

**Additional file 1.** **Table S1.** The sequences of the primers.

## Data Availability

The datasets used in our study are available from the corresponding author on reasonable request.

## Abbreviations

TC: Total cholesterol
LDL-C: Low-density lipoprotein cholesterol
TG: Triglycerides
HDL-C: High-density lipoprotein cholesterol
CVD: Cardiovascular disease
MiRNA: MicroRNA
HDL: High-density lipoprotein
CYP7A1: Cytochrome P450 7A1
PI3K: Phosphatidylinositol 3-kinase
SREBP-1: Sterol regulatory element-binding protein-1
SREBP-2: Attenuating sterol regulatory element-binding protein-2
SR-B1: Scavenger receptor type B1
PPARγ: Peroxisome proliferator-activated receptor-gamma
LPL: Lipoprotein lipase
Runx2: Runt-related transcription factor 2
CREB1: CAMP-response element binding protein1
LPGAT1: Lysophosphatidylglycerol acyltransferase 1
CRTC2: Coactivator 2
PPARGC1A: Peroxisome proliferator activated receptor gamma coactivator 1 alpha
